# BRD4 Inhibition Enhances Azacitidine Efficacy in Acute Myeloid Leukemia and Myelodysplastic Syndromes

**DOI:** 10.3389/fonc.2019.00016

**Published:** 2019-01-29

**Authors:** Fernando Vieira Pericole, Mariana Lazarini, Luciana Bueno de Paiva, Adriana da Silva Santos Duarte, Karla Priscila Vieira Ferro, Fernanda Soares Niemann, Fernanda Marconi Roversi, Sara Teresinha Olalla Saad

**Affiliations:** ^1^Hematology and Transfusion Medicine Center, Instituto Nacional de Ciência e Tecnologia do Sangue, University of Campinas, Hemocentro-Unicamp, São Paulo, Brazil; ^2^Department of Pharmaceutical Sciences, Federal University of São Paulo, São Paulo, Brazil; ^3^Universidade São Francisco (USF), Bragança Paulista, São Paulo, Brazil

**Keywords:** myelodysplastic syndromes, acute myeloid leukemia, BET member of bromodomain-containing proteins, azacitidine, AZD6738

## Abstract

Myelodysplastic syndromes (MDS) are clonal hematopoietic stem cell-based disorders characterized by ineffective hematopoiesis, increased genomic instability and a tendency to progress toward acute myeloid leukemia (AML). MDS and AML cells present genetic and epigenetic abnormalities and, due to the heterogeneity of these molecular alterations, the current treatment options remain unsatisfactory. Hypomethylating agents (HMA), especially azacitidine, are the mainstay of treatment for high-risk MDS patients and HMA are used in treating elderly AML. The aim of this study was to investigate the potential role of the epigenetic reader bromodomain-containing protein-4 (BRD4) in MDS and AML patients. We identified the upregulation of the short variant BRD4 in MDS and AML patients, which was associated with a worse outcome of MDS. Furthermore, the inhibition of BRD4 *in vitro* with JQ1 or shRNA induced leukemia cell apoptosis, especially when combined to azacitidine, and triggered the activation of the DNA damage response pathway. JQ1 and AZD6738 (a specific ATR inhibitor) also synergized to induce apoptosis in leukemia cells. Our results indicate that the BRD4-dependent transcriptional program is a defective pathway in MDS and AML pathogenesis and its inhibition induces apoptosis of leukemia cells, which is enhanced in combination with HMA or an ATR inhibitor.

## Introduction

Myelodysplastic syndromes (MDS) encompass a heterogeneous group of disorders characterized by molecular alterations in hematopoietic stem cells, leading to ineffective hematopoiesis and risk of acute leukemia. Progression to acute myeloid leukemia (AML) occurs in one third of the patients, and it is believed to be the result of increased genomic instability (1). The disease predominantly affects older patients, with a median age of around 70 years and annual incidence in the range of 4–8 cases/100,000 individuals (2). Elderly AML (>65 years) presents a median survival of just 5–6 months (3). MDS and AML cells exhibit aberrant methylation in promoter regions of tumor-suppressor genes, which has been tracked to the most immature cells (4, 5) and is associated to gene mutations involving epigenetic modifiers (6, 7). Increased DNA damage and alterations in the DNA damage response pathway (DDR) are critical features of gene instability that are implicated in the pathogenesis of MDS and AML (8).

Hypomethylating agents (HMA), especially azacitidine, are the current mainstay for the treatment of advanced MDS and elderly AML, inducing hematological improvement, partial and complete responses, resulting in long-term survival (3, 9). However, hematological improvement occurs in only 30% of patients on HMA therapy (10). Therefore, drug combinations aiming to increase HMA efficiency are needed. Bromodomain containing proteins (BET proteins) are the most prominent group of epigenetic reader proteins, recognizing and binding to acetylated lysine residues within histone tails. BET proteins influence gene expression, cell-cycle regulation and maintain an association with chromatin throughout mitosis, facilitating “gene bookmarking.” As a member of the BET family, BRD4 bromodomain containing 4 (BRD4) acts by inducing the expression of growth-promoting genes and has been described as a therapeutic target for AML (11). BRD4 generates two major transcriptional variants: long and short. The C-terminal domain of the long isoform of BRD4 has been described as crucial for maintaining normal chromatin structure (12). Conversely, BRD4 short variant function is less known. It has been shown as an endogenous inhibitor of DDR signaling, recruiting the condensing II chromatin remodeling complex to acetylated histones (13).

Herein, we describe an increased expression of the short variant of BRD4 in MDS and AML patients and establish BRD4 short variant overexpression as a new independent MDS prognostic factor. The combination of a BRD4 inhibitor (JQ1) and the HMA, azacitidine, was more effective than azacitidine or JQ1 alone for inducing cell apoptosis. JQ1 and AZD6738 (a specific ATR inhibitor) also synergized to induce apoptosis, suggesting a role for the combination of BET inhibitors with HMA or DDR inhibitors in MDS and AML treatment.

## Materials and Methods

### Patient Samples

Bone marrow samples were collected from patients with MDS (*n* = 58), AML with MDS-related changes AML (AML-MRC) (*n* = 16), *de novo* AML (*n* = 34), and healthy donors (*n* = 24). All patients included in the study were untreated at the time of sample collection. MDS patients were classified according to 2016 World Health Organization (WHO) classification (14) and according to revised international prognostic staging system (R-IPSS) (15). The cytogenetic risk for MDS and AML was defined according to R-IPSS (15) and to the Medical Research Council cytogenetic classifications (16), respectively. Healthy donors' and patients' characteristics are described in Table 1. All healthy donors and patients signed informed consent forms under a local research protocol. This study was approved by the Institutional Ethical Review Board in accordance to the Helsinki Declaration.

**Table 1:** Characteristics of healthy donors and patients.

	**Number**
**HEALTHY DONORS**	
Gender (male/female)	17/7
Age (years), median (range)	36 (23–69)
**PATIENTS**	
**MDS**	58
Gender (male/female)	34/24
Age (years), median (range)	64 (16–90)
WHO 2016 classification	
MDS-RS/MDS-MLD (< 5% BM blasts)	5/31
MDS-EB1/MDS-EB2 (≥5% BM blasts)	11/11
IPSS-R	
Very low/low risk	7/24
Intermediate/high/very high risk	10/12/5
Cytogenetic risk^a^	
Very good/good	2/46
Intermediate	3
Poor/very poor	2/2
No growth	3
***de novo*** **AML**	34
Gender (male/female)	18/16
Age (years), median (range)	55 (17–93)
BM blasts (%), median (range)	68 (28–98)
Cytogenetic risk^b^	
Good	5
Intermediate/Poor	19/4
No growth	6
**AML-MRC**	16
Gender (Male/Female)	11/05
Age (years), median (range)	70 (36–81)
BM blasts (%), median (range)^c^	45 (11–75)
Cytogenetic risk^d^	
Good	0
Intermediate/Poor	8/8
No growth	0

*MDS, myelodysplastic syndromes; WHO, World Health Organization; MDS-RS, MDS with ringed sideroblasts; MDS-MLD, MDS with multilineage displasia; MDS-EB1, MDS with excess blast-1; MDS-EB2, MDS with excess blast-2; IPSS-R, revised International Prognostic Scoring System; AML, acute myeloid leukemia; BM, bone marrow; AML-MRC, acute myeloid leukemia with myelodysplastic related changes*.

a *In MDS cohort, karyotype findings included very good risk: -Y (n = 1), del(11q) (n = 1); good risk: normal (n = 46); intermediate: inv(9) and del(16q22) (n = 1), rob(14;14) (n = 1), inv(9) (n = 1); poor: −7 (n = 2); very poor: >3 abnormalities (n = 2)*.

b *In AML cohort, karyotype findings included good risk: t_(8;21)_ (n = 4), inv(16) (n = 1); intermediate risk: normal (n = 16), trisomy 8 (n = 1), and other abnormalities (n = 2); poor risk included complex karyotype (n = 2), del(5q) (n = 1), and others (n = 1)*.

c *BM blasts percentage included 2 patients with lower than 20% blasts due to acute erythroid leukemia*.

d *In AML-MRC cohort, karyotype findings included intermediate risk: trisomy 8 (n = 2), normal (n = 4), and other abnormalities (n = 2); poor risk included complex karyotype (n = 5), del(5q) (n = 1), monosomy 7 (n = 2)*.

### CD34^+^ Cell Separation

Bone marrow mononuclear cells were isolated by Ficoll-Paque (GE, Uppsala, Sweden) from diagnostic samples of AML patients and cord blood units (CBU) samples from full-term deliveries. After that, primary human CD34^+^ hematopoietic stem and progenitor cells (CD34^+^ cells) were selected using immunomagnetic activated cell sorting columns (Miltenyi Biotech, Auburn, CA, USA), obtaining purity of at least 90%.

### Cell Culture

A panel of human myeloid leukemia cell lines (HEL, HL60, K562, and U937) was cultured in Roswell Park Memorial Institute medium-1640 (RPMI) (Sigma) containing 10% FBS, glutamine (2 mM), penicillin (100 μg/mL), streptomycin (100 μg/mL), and amphotericin B (0.25 μg/mL). The cells were maintained in a humidified atmosphere at 37°C and 5% CO_2_ and the experiments were performed when they reached exponential growth.

### Chemical Reagents

A BRD4 specific inhibitor (JQ1) was kindly provided by Dr. James Bradner (Dana-Farber Cancer Institute, Harvard Medical School, Boston, MA). The compound was diluted in dimethylsulfoxide (DMSO) to a 10 mM stock solution and was added to the cells at final concentrations of 0.24 μM to 100 μM for 48 h. Azacitidine (AZA) was also diluted in DMSO to a 100 mM stock concentration and added to cells at final concentrations of 1–3 μM for 48 h. The AZA concentration range was chosen based on the established AZA treatment schedule for MDS patients (75 mg/m^2^/day), which achieved plasma levels in the range of 3–11 μM (17, 18). The dose of 1 μM was used for both drugs when JQ1 and azacitidine were combined. AZD6738 was obtained from Selleck Chemicals (Houston, TX) diluted in DMSO and added to the cells at final concentrations of 1–20 μM. GI_30_ (drug inhibition of 30%) was determined after 48 h of exposure and used in combination with 1 μM of JQ1 or azacitidine.

### Gene Expression Analysis

RNA was extracted using the RNeasy Plus Micro Kit (Qiagen, Valencia, CA, USA) and reversely transcribed into cDNA with the *RevertAid H Minus First Strand cDNA Synthesis Kit* (MBI Fermentas, St. Leon-Rot, Germany). The quantitative RT-PCR (qRT-PCR) reaction was run with SYBR Green Master Mix PCR (Fermentas) using the ABI 7500 Sequence Detection System (Applied-Biosystem, Foster City, CA, USA). The values of the relative quantification of gene expression was calculated through the equation 2^−ΔΔ*CT*^ (19). A negative no “template control” was included for each primer pair and the amplification specificity was verified using a dissociation curve at the end of each run. Three replicas were run on the same plate for each sample. Sense and antisense primers were designed to be complementary to the sequences contained in different exons. The following primers were used: BRD4 long variant (*BRD4L*), 5′- AAAGGACCTGAAAATCAAGAACATG-3′ and 5′-GAAGCTGTCGCTGGATGACTT-3′; BRD4 short variant (*BRD4S*) 5′-CTGACAGCGAAGACTCCGAAA-3′ and 5′-GCTATAGCTTGCTGGGAAGGAA-3′, hypoxanthine phosphoribosyltransferase (*HPRT*), 5′-GAACGTCTTGCTCGAGATGTGA-3′ and 5′-TCCAGCAGGTCAGCAAAGAAT-3′.

### Western Blotting

Equal amounts of protein were submitted to electrophoresis on SDS polyacrylamide gels under reducing conditions and the nitrocellulose membrane was blotted with specific antibodies. Polyclonal antibodies against cleaved PARP-1 (sc-56196), CDK6 (sc-56282), GAPDH (sc-32233), and ACTIN (sc-1616) were obtained from Santa Cruz Biotechnology (Santa Cruz, CA, USA). Anti-phospho-histone H2AX (Ser139) (pH2AX) (9718), P53 (2524), and anti-caspase 3 (9665) were purchased from Cell signaling (Danvers, MA, USA) and P21 were obtained from Abcam (Cambridge, MA, USA). The target proteins were analyzed by chemiluminescence using an ECL Plus Kit (GE-Healthcare, Buckinghamshire, England). Quantitative analyses of the optical intensities of protein bands were determined with UN-SCAN-IT graph digitalizing software (Silk scientific, UT, USA) and normalized by actin or GAPDH for protein expression.

### Assessment of Cell Growth

Cell growth was measured by methylthiazoletetrazolium (MTT) assay. Briefly, 2.5 × 10^4^ cells per well were plated in 96–well plates in RPMI/10% FBS for 48 h. MTT solution (5 mg/mL) was added to each well and incubated at 37°C for 4 h. The reaction was stopped by 0.1 N HCl in anhydrous isopropanol. Cell growth was evaluated by measuring the absorbance at 570 nm, using an automated plate reader (Multiscan MS, Labsystems).

### Assessment of Apoptosis

Cell death was measured by annexin-V and PI assay. Briefly, the cells were seeded in 24-well plates and treated or not with different concentrations of JQ1 and/or azacitidine for 48 h. After this period, the cells were collected and incubated with 1 μg/mL propidium iodide (PI) and 1 μg/mL APC-Annexin-V for 15 min at room temperature in the dark. All specimens were analyzed on a FACSCalibur (BD Biosciences, CA, USA) and 10,000 events were acquired for each sample.

### Transduction With Lentivirus

U937 cells were transduced with lentivirus-mediated shRNA non-specific control (sc-108080) or lentivirus-mediated shRNA targeting BRD4 (sc-43639-V) from Santa Cruz Biotechnology (Santa Cruz Biotechnology, Santa Cruz, CA, USA) and named shControl and shBRD4 cells, respectively. Pooling of multiple BRD4 shRNAs (three different sequences) was used to reduce possible off-target effects of the shRNAs. BA/F3 cells were transduced with lentivirus particles produced using pCDH-MSV-MCS-EF1α empty vector (control) or containing the full lengths of BRD4S or BRD4L. Briefly, 2 × 10^5^ cells were transduced with lentivirus by spinoculation at multiplicity of infection (MOI) equal to 1 and selected with specific antibiotics for at least 7 days before using in the experiments. BA/F3 cells were also sorted using a FACsAria Fusion (Becton–Dickinson Biosciences) in addition to antibiotic selection.

### Cell Cycle Analysis

Cells were seeded on a 24-well plate and treated or not with different concentrations of JQ1 for 48 h. Next, the cells were collected and fixed overnight in 70% ethanol. DNA was stained with Pipes buffer containing PI (20 μg/mL) and RNase A (10 μg/mL). Cell fluorescence was detected with a FACSCalibur. The proportions of cells in the cell cycle phases were analyzed by Modifit (Verify Software House Inc., Topsham, ME, USA), according to DNA distributions.

### Statistical Analysis

Statistical analysis was performed using GraphPad Prism5 software (GraphPad Software, Inc., San Diego, CA, USA) or SAS System for Windons 9.2 (SAS Institute, Inc., Cary, NC, USA). Data were expressed as the median [minimum-maximum]. For comparisons, an appropriate Mann–Whitney test, Student's *t*-test or ANOVA was used. Comparison between patient groups was performed by analysis of *t*-test and covariance (ANCOVA) controlled for age, followed by *post-hoc* comparisons using the Tukey test. All experiments were repeated at least four times. Cox regression model was used to estimate overall survival (OS) and event-free survival (EFS) for MDS patients. The stepwise process of selection was used for multivariate analysis. OS was defined as the time (in months) between the date of sampling and the date of death (for deceased patients) or last follow-up (for censored patients). EFS was defined as the time (in months) between the date of sampling and the first event (death or MDS progression or leukemic transformation) or last follow-up (for censored patients). All tests were two-tailed. *P* ≤ 0.05 were considered statistically significant.

## Results

### *BRD4* Short Variant Expression Is Increased in Total Bone Marrow Cells From MDS and AML Patients and Associates With Worse Outcomes in MDS

The first step of this study comprised the evaluation of mRNA levels of both *BRD4* variants in total bone marrow cells from healthy donors (*n* = 24), MDS (*n* = 58), and AML (*n* = 50) patients. In order to exclude confounders, we carried out an ANCOVA analysis, which showed that age and gender did not interfere in our results.

**Figure 1:**
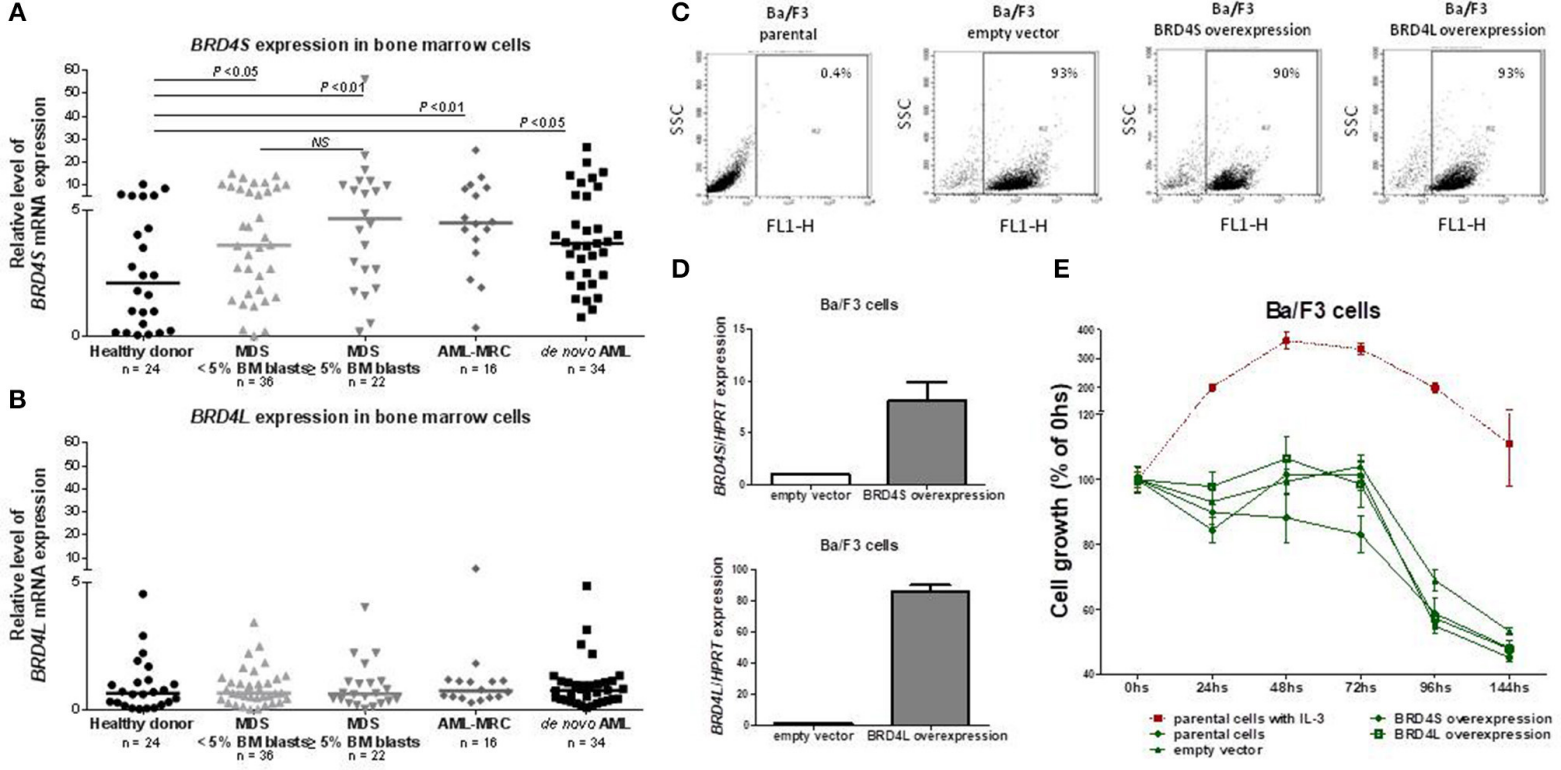
*BRD4* short variant gene is overexpressed in MDS and AML patients. *BRD4S* mRNA expression in total bone marrow cells from healthy donors, MDS <5% BM blasts, >5% BM blasts, AML-MRC and *de novo* AML patients **(A)**; *BRD4L* mRNA expression in total bone marrow cells from healthy donors, MDS <5% BM blasts, >5% BM blasts, AML-MRC, and *de novo* AML patients **(B)**; Efficiency of GFP-positive BA/F3 cells transduced with empty vector, *BRD4S* and *BRD4L*, in that order, measured by flow cytometry **(C)** and by quantitative PCR **(D)**; **(E)** Comparative growth (normalized by the initial number of cells) of BA/F3 parental cells (with and without IL-3), control (empty vector) or transduced with full-length *BRD4S* or *BRD4L*. The number of subjects and significant *P-*values (Mann–Whitney test) are indicated in the graph. MDS, myelodysplastic syndromes; AML, acute myeloid leukemia; AML-MRC, acute myeloid leukemia with myelodysplastic related changes.

*BRD4S* expression was significantly increased in both MDS (4.21 [0.01–56.17]) and AML (4.01 [0.33–26.58]) patients, when compared to healthy donors (2.11 [0.04–10.32]; all *P* < 0.01) (Figure 1A). No difference in *BRD4L* expression was observed between healthy donors, MDS and AML patients (Figure 1B). There were no differences when MDS patients were stratified according BM blasts or when AML patients were grouped into *de novo* AML or AML with myelodysplasia related changes (AML-MRC).

With a median follow-up time of 34.4 months, *BRD4S* expression appeared as one of the variables with significant impact in event-free survival (EFS) and overall survival (OS) of MDS patients in univariate analysis. The bone marrow blast percentages (absolute values) and R-IPSS (stratified into very low/low, intermediate and high/very high) also significantly altered EFS and OS. Multivariate analysis demonstrated that higher *BRD4S* expression, along with intermediate and high/very high R-IPSS risk MDS, were the only independent factors related to worse outcomes (Table 2).

**Table 2:** Univariate and multivariate analysis for event-free survival and overall survival of MDS patients.

**Factor**	**Univariate analysis**						**Multivariate analysis**					
	**Event free survival**			**Overall survival**			**Event free survival**			**Overall survival**		
	**HR**	**95% C.I**.	***P***	**HR**	**95% C.I**.	***P***	**HR**	**95% C.I**.	***P***	**HR**	**95% C.I**.	***P***
**IPSS-R RISK GROUP**												
Intermediate vs. very low/low	6.0	2.2–16.6	**0.0006**	6.9	2.4–20.0	**0.0004**	4.9	1.6–15	**0.006**	4.8	1.6–14.7	**0.007**
Very high/high vs. Very low/low	8.9	3.4–22.7	**<0.0001**	12.4	4.6–33.6	**<0.0001**	9.7	3.4–27.8	**<0.0001**	12.3	4.2–35.7	**<0.0001**
**BM BLAST PERCENTAGE**												
Absolute values	1.1	1.1–1.2	**<0.0001**	1.2	1.1–1.2	**<0.0001**						
***BRD4L*** **EXPRESSION**												
Absolute values	1.2	0.8–1.8	0.34	1.2	0.8–1.8	0.75						
***BRD4S*** **EXPRESSION**												
Absolute values	1.05	1.01–1.08	**0.02**	1.05	1.01–1.09	**0.02**	1.04	1.01–1.09	**0.02**	1.05	1.01–1.10	**0.01**

*Statistically significant P-values are highlighted in bold; MDS, myelodysplastic syndromes; HR, hazard ratio; when HR > 1 indicates that the first factor has the poorer outcome; C.I, confidence interval; BM, bone marrow; BM, blast percentage, BRD4L and BRD4S expression were analyzed as continuous numerical values*.

Aiming to explore a possible function of BRD4 as an oncogene, we overexpressed *BRD4L* or *BRD4S* using GFP tagged in normal hematopoietic murine cells (BA/F3), which are dependent on IL-3, and accessed daily the cell growth for 144 h in the presence or absence of 20 ηg/mL IL-3. The overexpression of both variants was effective (Figures 1C,D), but overexpression of BRD4L or BRD4S did not lead to IL-3 independency (Figure 1E). In the presence of IL-3, the growth of BRD4S or BRD4L overexpressed cells were also similar to the control (data not shown). These results suggest that BRD4 does not act as an oncogene alone.

### JQ1 Treatment Reduces Cell Viability, Along With the Induction of Apoptosis and G0/G1 Cell Cycle Arrest in Myeloid Leukemia Cell Lines, but Not in Normal CD34^+^ Cells

**Figure 2:**
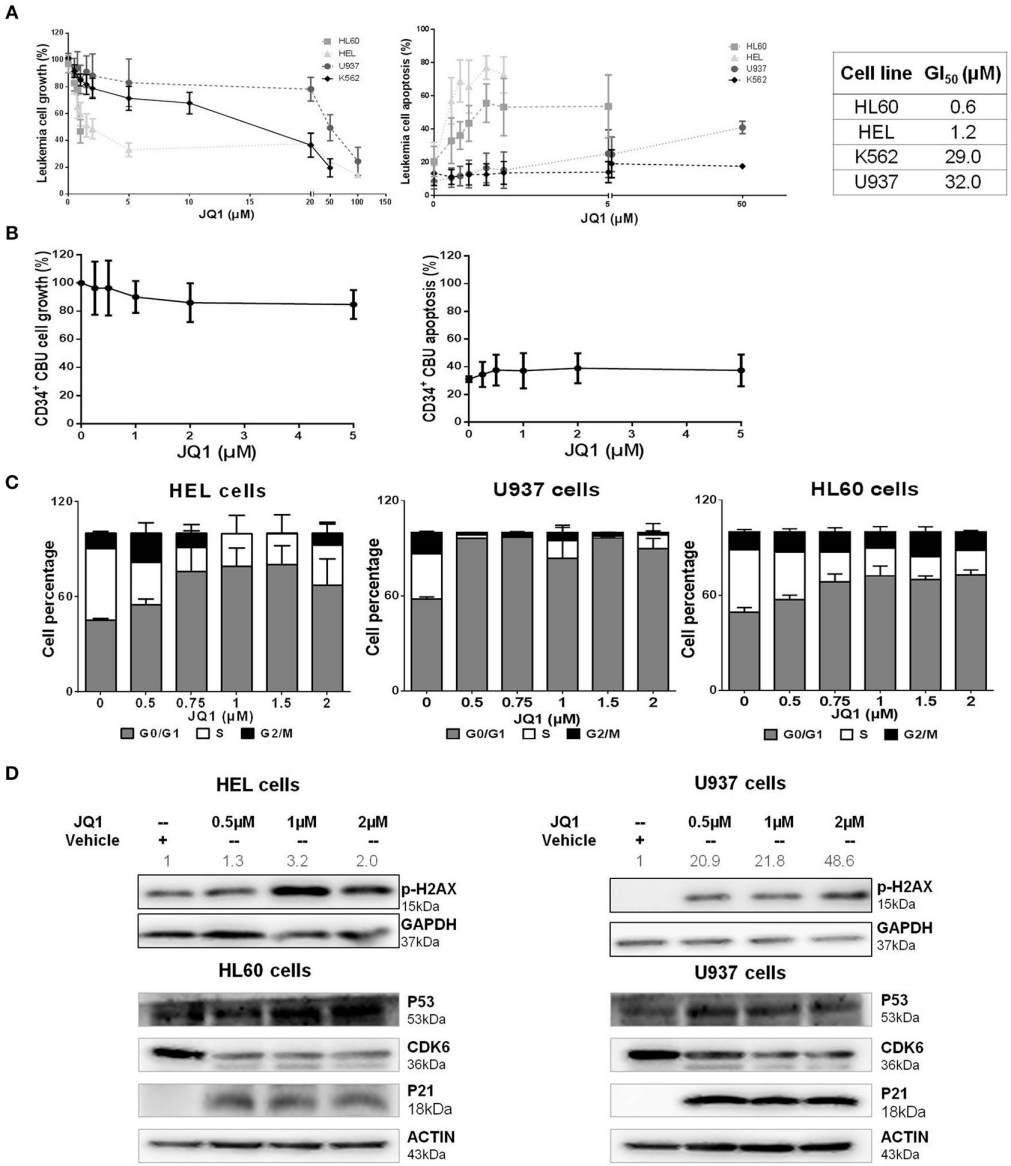
JQ1, a BRD4 inhibitor, reduces cell viability, induces apoptosis and cell cycle arrest of leukemia cell lines, with variable drug sensitivity. **(A)** Cell viability and apoptosis were determined by MTT assays and flow cytometry after 48 h of JQ1 treatment and growth inhibition (GI_50_) of four leukemia cell lines (HL60, HEL, K562, and U937). GI_50_ values determined from at least five independent experiments, by non-linear curve fitting to a sigmoid dose-response model; **(B)** Treatment of CD34^+^ cells from cord blood units (CBU) with increasing doses of JQ1 showed no significant cell viability reduction or apoptosis induction in that order; **(C)** Cell cycle phases were detected by flow cytometry. The cell lines tested are indicated in the graph. Results are shown as the percentage of total cells and bars represent increasing doses of JQ1. Graph bars represent a minimum of 6 independent experiments. **(D)** Western blotting analysis of total cell extracts from HEL, HL60, and U937 cells after 48 h of treatment with JQ1. The membrane was blotted anti-pH2AX (15 kDa), P53 (53 kDa), CDK6 (36 kDa), P21 (18 kDa), and for GAPDH (37 kDa) or ACTIN (43 kDa) as a control for equal sample loading, and developed with the ECL Western Blotting Analysis System. Densitometry was performed and the ratio of p-H2AX vs. GAPDH compared with the normalized value of control is shown. Western blot figures are representative of all experiments performed. All bar graphs represent mean ± *SD* of at least five independent experiments.

In order to characterize the role of BRD4 in the disease pathogenesis, a panel of myeloid leukemia cell lines was treated with JQ1, an inhibitor of both BRD4 isoforms and other BET protein members (20, 21). Treatment with JQ1 induced a dose-dependent reduction in cell viability, in association with a similar induction of apoptosis. U937 and K562 cells were more resistant to the treatment, and presented higher GI_50_ (growth inhibitory concentration, with 50% reduction in cell viability) levels compared with HL60 and HEL cells (Figure 2A). Normal CD34^+^ cells from four samples of cord blood units exhibited greater tolerability to JQ1, with sustained cell viability and lower apoptosis rate compared to neoplastic cells (Figure 2B). JQ1 treatment induced an increase in G0/G1 cell cycle in cell lines, indicating cell cycle arrest (Figure 2C). We also observed an increase in p-H2AX in both HEL (more sensitive) and U937 (more resistant) cell lines, along with decreasing levels of CDK6 and with higher P53 and P21 protein concentrations (Figure 2D). This result suggests that BRD4 pharmacological inhibition causes DDR pathway activation and cell cycle arrest, even when the cells were exposed to a lower dose of JQ1.

### JQ1 Exhibits an Additive Effect Together With Azacitidine on the Apoptosis of Leukemia Cell Lines and CD34^+^ AML Primary Cells

**Figure 3:**
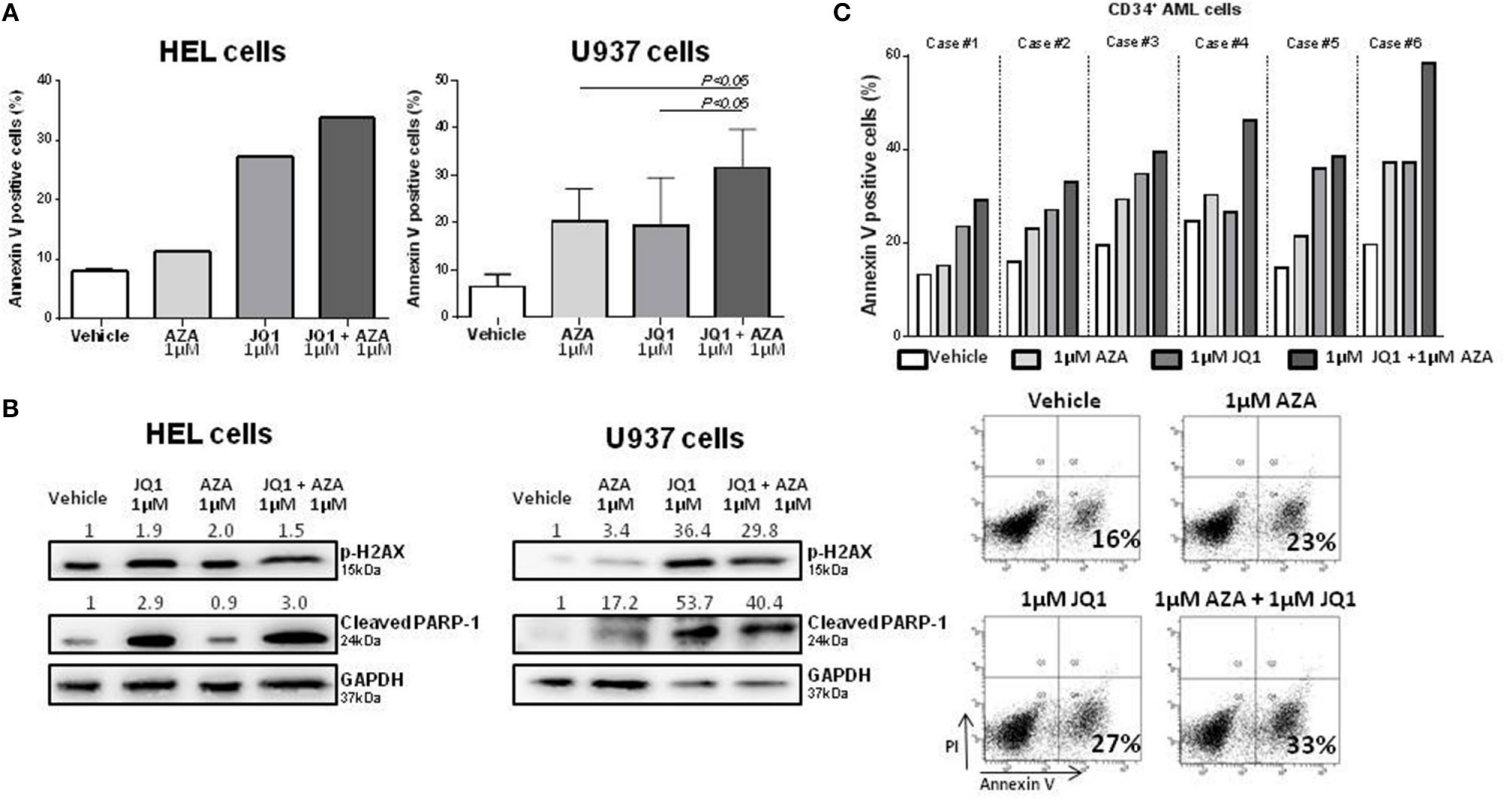
Combined treatment of JQ1 and azacitidine induces an additive effect on apoptosis of HEL, U937, and primary AML cells.**(A)** Apoptosis rate of HEL and U937 cell lines treated with JQ1, AZA, or JQ1+AZA for 48 h: Bar graphs show mean ± *SD* of at least four independent experiments. **(B)** Western blotting of HEL and U937 cell lines extract showing increased p-H2AX and cleaved PARP expression after JQ1+AZA treatment. Densitometry was performed and the ratio of target proteins vs. GAPDH compared with the normalized value of control is shown. **(C)** Apoptosis rate of 5 primary CD34^+^ AML cells treated with JQ1, AZA, and JQ1+AZA and a representative plot showing increasing apoptosis under JQ1+AZA combination.

With the aim of further characterizing the potential combinatory effects of BRD4 inhibition and a standard treatment for MDS, HEL, and U937 cells were treated with JQ1 and azacitidine for 48 h. JQ1 + AZA treatment increased the apoptotic rate of both cell lines, particularly in U937 cells (Figure 3A). JQ1 treatment increased p-H2AX and cleaved PARP-1, suggesting that activation of DDR is an important mechanism for apoptotic induction after JQ1 treatment. Although annexin V positive cells were increased with the co-treatment, we did not observe the same synergism in pH2AX and cleaved PARP-1 expression, possibly because high levels of these proteins were already detected when JQ1 was used alone (Figure 3B). CD34^+^ cells were isolated from diagnostic samples of 5 AML patients and treated with JQ1, AZA or both. In all samples, the combination of JQ1 and AZA induced a higher apoptosis rate than JQ1 or AZA alone (Figure 3C).

### *BRD4* Silencing Decreases Proliferation and Apoptosis and Potentiates the Proapoptotic Effect of Azacitidine

**Figure 4:**
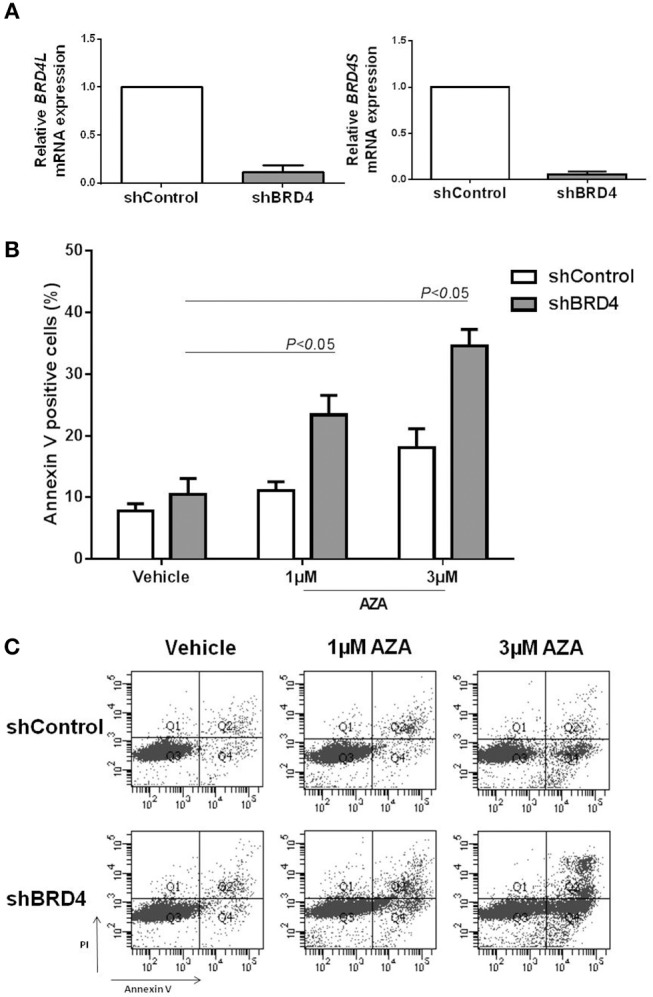
BRD4 silencing increases apoptosis rate of U937 cells under azacitidine treatment. **(A)** Efficacy of *BRD4* silencing in U937 cells transduced with lentivirus-mediated control (shControl) or BRD4 (shBRD4) measured by RT-PCR. **(B)** Silenced and control cells were treated for 48 h with vehicle (DMSO) or azacitidine (1 or 3 μM): and apoptosis was detected by flow cytometry. **(C)** Representative plot of increasing apoptosis of shBRD4 cells under AZA treatment. Significant *P*-values (one-way Anova test) are indicated in the graph. All bar graphs represent mean ± *SD* of at least four independent experiments.

We next sought to investigate whether *BRD4* gene silencing would produce similar results to that of JQ1 treatment, in order to exclude possible therapy-related off target effects. For this purpose, U937 cells were stably transduced with lentivirus-mediated shRNA targeting BRD4 (shBRD4) or an appropriate control (shControl) and the efficacy of the transduction was confirmed by qRT-PCR (Figure 4A).

Apoptosis was evaluated in silenced cells treated or not with 1 or 3 μM AZA for 48 h. Following AZA treatment, shBRD4 cells showed a significant increase in apoptotic rate (*P* < 0.05) (Figures 4B,C), similarly to that observed for the drug combination.

### JQ1 Potentiates the Effects of the ATR Inhibitor (AZD6738) on the Apoptosis of Leukemia Cells

**Figure 5:**
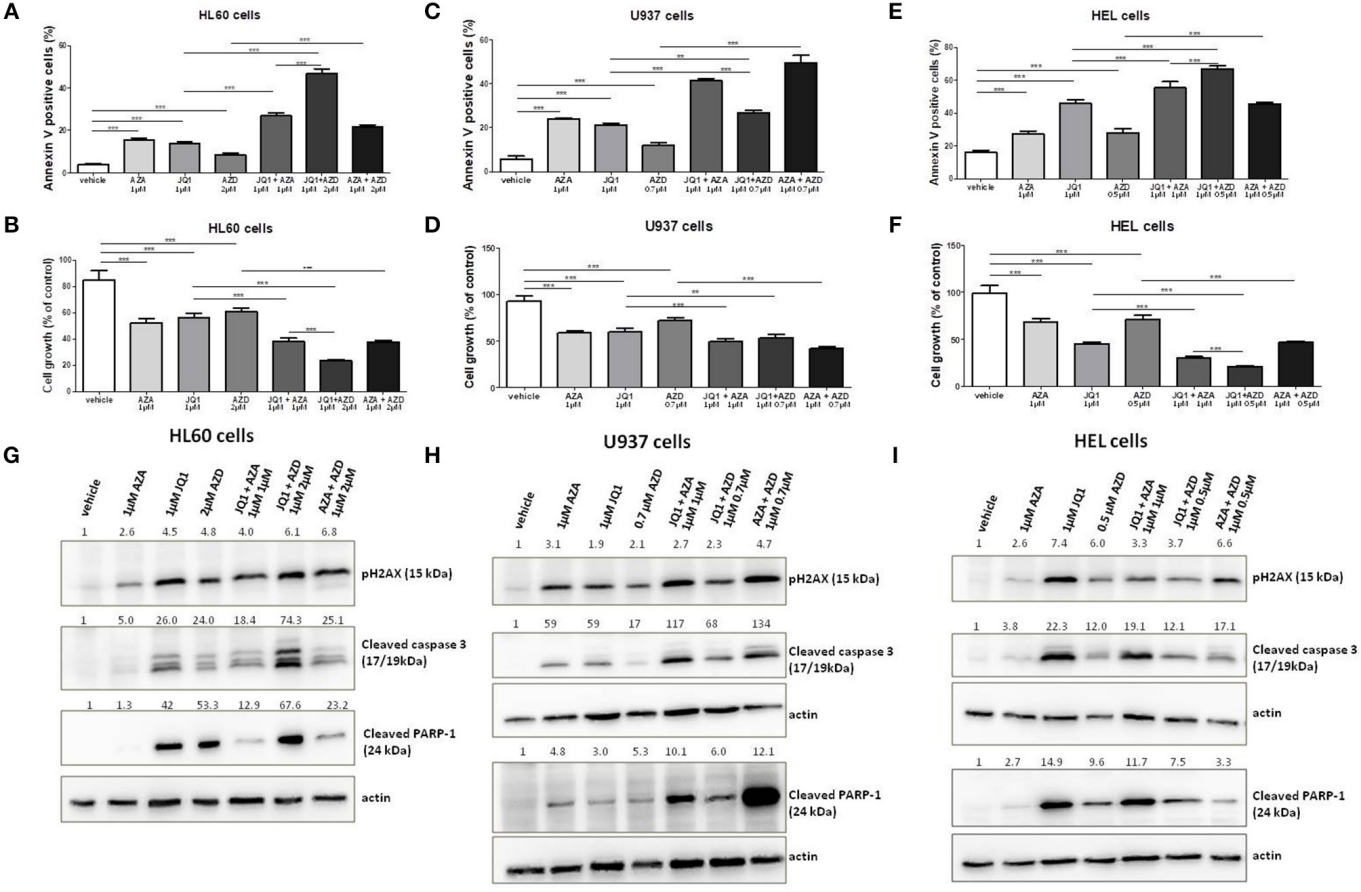
JQ1 effects on apoptosis of leukemia cell lines are increased with the ATR inhibitor AZD6738. Apoptosis rate and cell growth percentage in HL60 **(A,B)**, U937 **(C,D)**, and HEL **(E,F)** leukemia cell lines treated with AZA, JQ1, or AZD6738 monotherapy or in double combinations in the indicated doses for 48 h. Protein expression levels of pH2AX, cleaved caspase 3, and cleaved PARP-1 in HL60 **(G)**, U937 **(H)**, and HEL **(I)** leukemia cell lines treated with AZA, JQ1, or AZD6738 monotherapy or in double combinations in the indicated doses for 48 h. Actin (42 kDa) was used as a control for equal sample loading and the membrane was developed with the ECL Western Blotting Analysis System. Densitometry was performed and the ratio of target proteins vs. actin compared with the normalized value of control is shown. All bar graphs represent mean ± SD of at least four independent experiments. ^**^P ≤ 0.01; ^***^P ≤ 0.001.

Ataxia telangiectasia and Rad3 related (ATR) inhibitors have been reported to display synergism with JQ1 in the induction of apoptosis in lymphoma and melanoma cells (22, 23). Therefore, we aimed to test and compare the effects of JQ1 combined with a specific ATR inhibitor (AZD6738) or with azacitidine in leukemia cells. The GI_30_ of AZD6738 was firstly calculated in HEL, U937, and HL60 cell lines (Figure S1). Subsequently, these cell lines were exposed to 1 μM JQ1, 1 μM azacitidine or the GI_30_ dose of AZD6738 monotherapy or double combined treatment courses (JQ1 + azacitidine, JQ1 + AZD8768, azacitidine + AZD6738) for 48 h. In addition to U937 and HEL cells, a synergic effect of JQ1 and azacitidine was observed in HL60 cells (Figures 5A–F). In HL60 cells, the effects of JQ1 + AZD6738 were stronger than JQ1 + azacitidine (Figure 5A), whereas JQ1 + azacitidine was more potent in U937 cells (Figure 5C) and both combinations produced similar effects in HEL cells (Figure 5E). AZA + AZD6738 was also a promising combination, especially in U937 cells (Figure 5B). pH2AX was induced by all treatment schemes (monotherapy or combinations) in the three cell lines (Figures 5G–I). Cleaved caspase-3 expression was consistent with annexin V and cleaved PARP-1 results (Figures 5G–I).

## Discussion

Our results suggest that the short variant of BRD4 is a biological determinant of MDS phenotype and aggressiveness, since the two MDS subtypes (with lower and higher blast percentages) and AML patients exhibited overexpression of *BRD4S*, but particularly those with higher-risk disease. Moreover, a higher expression of this gene predicted a worse overall outcome in our cohort, in accordance with similar results for BRD4 overexpression and worse outcomes in solid tumors (24–26).

The overexpression of BRD4 isoforms in BA/F3 cells did not induce IL-3 independence, suggesting that this gene it is not and oncogene by itself. Moreover, *in vitro* studies showed that *BRD4* inhibition, combined with azacitidine or AZD6738, synergistically induced apoptosis of leukemia cells.

Floyd et al. (13) described the BRD4 short isoform as an endogenous inhibitor of DNA damage response since cell lines with forced BRD4 hyper expression exhibited attenuated DNA damage response signaling (13). In this context, we speculate that the BRD4 short isoform could decrease the DNA damage response, favoring the disease toward genetic instability and clonal evolution. Our results using the BA/F3 cell model indicate that BRD4 does not function as an oncogene when overexpressed alone, which is in accordance with previous studies that have demonstrated that BET bromodomain proteins are not oncogenes themselves, but may act as a coactivators or direct modulators of other oncogenes, such as MYC and E2F proteins (27, 28).

In accordance with experimental data from other cancers, our data showed that BRD4 inhibition leads to variable apoptosis induction. However, since BRD4 also causes an arrest in G0/G1, and cells unable to differentiate undergo apoptosis, we cannot affirm that BRD4 acts directly on apoptosis. Importantly, we observed an additive effect of azacitidine combined with either JQ1 treatment or *BRD4* silencing. To our knowledge, this is the first evidence for an additive effect between the standard treatment for MDS and elderly AML (the hypomethylating agent azacitidine) and a drug targeting an epigenetic modulator other than histone deacetylase.

Interestingly, JQ1 treatment increased the levels of p-H2AX, indicating a DDR pathway activation, even when using a lower dose. Double strand-breaks (DSB) in damaged DNA cause the phosphorylation of the neighboring histone H2AX at Ser139 via ATM. The functional significance of p-H2AX is a signal that facilitates DSB repair, presumably by causing the chromatin to be more accessible for DNA repair. Once DNA repair fails, the cells undergo death by activating apoptosis. Thus, apoptosis induction following DNA damage is a protective mechanism that prevents carcinogenesis (29). Some recent evidence suggests that DNA methyltransferase 1 (DNMT1) inhibition could also activate DDR, reinforcing the idea that epigenetic and genetic stability are intrinsically linked to one another (30, 31). JQ1 is a Brodomain and Extra-terminal (BET) inhibitor with activity blocking BRD4 (short and long isoforms), but also against other BET protein members, such as BRD2, BRD3, and BRDT. Therefore, this non-selective effect of JQ1 needs to be taken into consideration in the interpretation of the data.

Despite not having yet been analyzed specifically in MDS patients, there is increasing evidence supporting a role for BRD4 in AML pathogenesis and its potential therapeutic applicability. Herrmann et al. (32) described the overexpression of BRD4 in AML patient samples, even in highly enriched CD34^+^/CD38^−^ and CD34^+^/CD38^+^ stem and progenitor cells and showed that JQ1 was capable of inducing the apoptosis of these cells. They also demonstrated that JQ1 synergized with cytarabine, reducing the cell viability of AML cells. Chen et al. (33) showed that BRD4 inhibition induced differentiation and death of IDH2 mutated AML, whereas Dawson et al (34) reported a role for NPM1 mutation in the induction of a BRD4 transcriptional program and that BET inhibition restored the NPM1 nuclear localization and abrogated the BRD4-induced oncogenic transcriptional program.

Stewart et al. (35) showed that JQ1 causes caspase 3/7-mediated apoptosis and a DNA damage response in DNMT3a/NPM1-mutated AML, suggesting that JQ1 might sensitize AML cells to p53-mediated cell death. Moreover, recent data suggest a synergistic effect of the histone deacetylase inhibitor, panobinostat, and JQ1 in AML cells (36). DDR activation seems to function as a biomarker of BRD4 inhibition efficacy, as cell lines presenting DDR activation also showed apoptosis induction. Chen et al. (37) recently showed that AZA-resistant MDS/AML cells have significantly increased expressions of BRD4, BRD2, and DNMT1. However, many DNA damage-induced cell death pathways are active in mammalian cells and the involved mechanisms and protein networks are complex and not fully understood (38). Therefore, our study indicates (not confirm) that BRD4 inhibition induces apoptosis through the activation of DDR.

Li et al. (39) recently demonstrated similar results regarding the role of BRD4 in prostate cancer, where higher gene expression was associated with worse outcomes and BRD4 inhibition activated H2AX. The authors showed that BRD4 is essential for the repair of DNA double-strand breaks (39). Muralidharan et al showed a synergistic effect of BRD4 inhibition with ATR inhibitors, inducing DDR in lymphoma cell lines (23), as demonstrated here in leukemia cells.

Larger and different cohorts of patients are necessary to confirm our results, since our cohort had low number of high-risk cytogenetics patients. Our findings however are in accordance with those from other authors supporting the existence of a defective BRD4-dependent transcriptional program in MDS and AML. The *in vivo* efficacy of JQ1 or other BET-targeting drugs for MDS patients is currently unknown, even though preliminary experimental and clinical data from BET inhibition in AML patients showed that BET-targeting drugs presented low toxicity profile and promising efficacy. Importantly, cord blood CD34 cells tolerated the effects of BRD4, with sustained cell viability and lower apoptosis, suggesting that BRD4 has a different role in normal vs. leukemic progenitor cells and, therefore, leukemic cells could be more BRD4 dependent than normal progenitor cells.

In conclusion, the BRD4 short variant is upregulated in MDS and AML patients and functions as an independent MDS prognostic factor, predicting worse outcomes. Our *in vitro* results further demonstrated that BRD4 inhibition shows a synergism with azacitidine or AZD6738, activating apoptosis, possibly through DDR pathway activation.

## Author Contributions

FP designed and performed the experiments, participated in patient follow-up, collected, analyzed, and interpreted the data, and wrote the manuscript. ML performed experiments, analyzed, and interpreted data and wrote the manuscript. LdP performed experiments. FR contributed to the experiments and analyzed and interpreted data. AD, KV, and FN performed experiments. SO provided the study conception, directed the research, provided financial support, revised, and gave final approval of the manuscript.

### Conflict of Interest Statement

The authors declare that the research was conducted in the absence of any commercial or financial relationships that could be construed as a potential conflict of interest.

## Abbreviations

MDS: myelodysplastic syndromes
AML: acute myeloid leukemia
BET: bromodomain containing proteins
HMA: hypomethylating agents
BRD4: Bromodomain-containing protein 4
AZA: azacitidine.
