# Supplementary material for: BRD4 Inhibition Enhances Azacitidine Efficacy in Acute Myeloid Leukemia and Myelodysplastic Syndromes
Source: Front Oncol. 2019 Jan 29;9:16. doi: 10.3389/fonc.2019.00016 (PMC6361844; doi:10.3389/fonc.2019.00016)
Supplement: Supplementary file 1 [file Data_Sheet_1.doc]

**Supplementary Material**

**BRD4 inhibition enhances azacitidine efficacy in acute myeloid leukemia and myelodysplastic syndromes**

Fernando Vieira Pericole^1^, Mariana Lazarini^1,2^ Luciana Bueno de Paiva^1^, Adriana da Silva Santos Duarte^1^, Karla Priscila Ferro^1^, Fernanda Niemman^1^, Fernanda Marconi Roversi^1^ Sara Teresinha Olalla Saad^1^.

## ^1^ Hematology and Transfusion Medicine Center-University of Campinas/Hemocentro-Unicamp, Instituto Nacional de Ciência e Tecnologia do Sangue, Campinas, São Paulo, Brazil.

^2^ Department of Pharmaceutical Sciences, Federal University of São Paulo, Diadema, São Paulo, 09913-030, Brazil

Corresponding author:

Sara T. Olalla Saad

Hematology and Transfusion Medicine Center - University of Campinas (UNICAMP), Carlos Chagas, 480 - Cidade Universitária Zeferino Vaz - Barão Geraldo, Campinas, São Paulo, Brazil. CEP: 13083-878. Phone: +55 19 35218665 and Fax: +55 19 32891089.

e-mail: sara@unicamp.br

This file contains:

Supplementary Figure S1

**
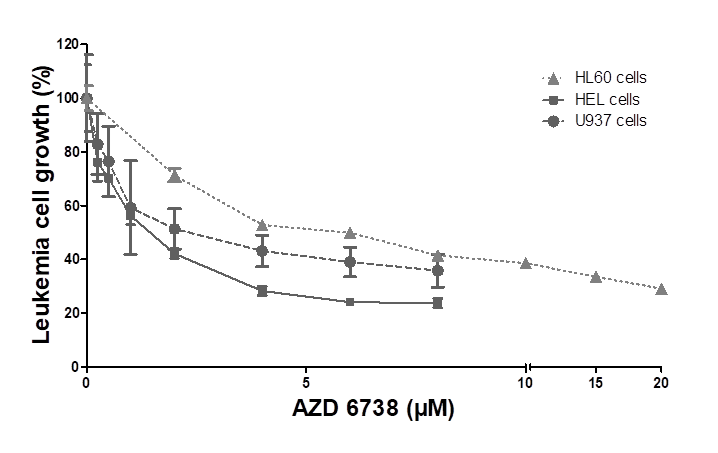
**

**Figure S1: Cell viability of leukemia cell lines treated with AZD6738.** Cell viability of three leukemia cell lines (HL-60, HEL and U937) was determined by MTT assays after 48h of treatment with AZD6738. Results represent media and standard deviation from three independent experiments. GI_30_ (growth inhibitory concentration, with 30% reduction in cell viability) were calculated and used in further experiments.
